# Pan-cancer analysis of the FAM83 family and its association with prognosis and tumor microenvironment

**DOI:** 10.3389/fgene.2022.919559

**Published:** 2022-07-22

**Authors:** Shangkun Yuan, Zhisheng Huang, Xiaoying Qian, Yong Wang, Chen Fang, Renfang Chen, Xinwei Zhang, Zhehao Xiao, Qian Wang, Biao Yu, Yong Li

**Affiliations:** ^1^ Department of Medical Oncology, The First Affiliated Hospital of Nanchang University, Nanchang, China; ^2^ Medical Innovation Center, The First Affiliated Hospital of Nanchang University, Nanchang, China; ^3^ Graduate School of Peking Union Medical College, Chinese Academy of Medical Sciences, Peking Union Medical College, Beijing, China

**Keywords:** FAM83 family, pan-cancer, prognosis, tumor microenvironment, bioinformatics

## Abstract

Family with sequence similarity 83 (FAM83) is a newly identified family of oncogenes whose members play important roles in signaling and cancer progression. However, a thorough understanding of the FAM83 family in tumors is still lacking. Here, we performed a comprehensive analysis of the expression levels of the FAM83 family across cancers and patient prognoses using bioinformatics methods. We found that the expression levels of FAM83 family genes were upregulated in most tumors, and importantly, high expression levels of FAM83 family genes were related to poor prognosis in most tumors. In addition, we analyzed the relationship of FAM83 family genes with immune subtypes and the tumor microenvironment (TME). The results showed that FAM83 family genes were significantly associated with immune infiltrative subtypes and to varying degrees with the level of stromal cell infiltration and tumor stem cells. Finally, our study also showed the relationship between FAM83 family genes and drug sensitivity. Therefore, this pan-cancer analysis demonstrates the critical role of FAM83 family genes in tumor development and provides new clues for therapeutic strategies for cancer.

## Introduction

Cancer is a major public health problem and the second highest cause of death globally. The incidence and mortality of cancer also continue to grow, seriously threatening human health (Chen et al., 2015; Siegel et al., 2021). Therefore, it is of important significance to study tumor invasion, drug resistance, and the tumor microenvironment to determine novel therapeutic targets and methods of tumor control.

In recent studies, FAM83 family genes have been shown to be remarkably upregulated in multiple cancer types in humans (Snijders et al., 2017). The FAM83 protein family consists of eight members (A–H), each of which is situated at a different genomic locus (Bartel et al., 2016). Every FAM83 gene codes for a protein that is categorized solely based on the existence of a well-conserved unknown domain (DUF1669) situated at the N-terminal (Ma et al., 2021). However, there is a unique variably length C-terminal for each member, whose biological function and associated mechanisms might differ (Bozatzi and Sapkota, 2018; Fulcher et al., 2018). Increasing evidence suggests that the FAM83 family plays an essential role in tumor signaling and cancer development, and thus, it is emerging as an interesting oncogene worthy of further investigation (Lin et al., 2021; Zhang et al., 2021).

Initially, Bissell discovered that FAM83A is overexpressed in breast cancer, which can lead to EGFR-TKI resistance and promote tumorigenesis by interacting with c-RAF and PI3K p85 (Lee et al., 2012), and in the same year, Jackson Laboratories identified FAM83B as an oncogenic transformation driven by EGFR-RAS-MAPK mediators (Cipriano et al., 2012). Since then, several studies have progressively demonstrated that the FAM83 family of genes is critical in tumor growth, invasion and chemical resistance. For example, FAM83A was found to activate various signaling pathways, such as PI3K/ATK, Wnt, and Hippo, accelerating the progression of lung cancer (Zhou et al., 2019; Hu et al., 2020; Zheng et al., 2020). Moreover, FAM83B suppresses cisplatin resistance and slows ovarian tumor progression (He et al., 2021). Similarly, it has been found that FAM83D activates the MEK/ERK and Wnt/β-catenin pathways to enhance cell proliferation (Mu et al., 2017; Wang et al., 2019a). In addition, compared to paracarcinoma tissues, high expression of FAM83H in cancer tissues has been associated with poor prognosis in cancers of the pancreas (Zhuang et al., 2020) and bones (Kim et al., 2019), but FAM83H has been related to good survival in cancers of the head and neck (Snijders et al., 2017). Thus, the oncogenic effects of the FAM83 family gene may vary in various tumors. Although current studies show that the FAM83 family plays an important role in cancer, the association between the FAM83 gene and the tumor microenvironment and drug sensitivity remains unreported. Therefore, the mechanics of the role of FAM83 family genes in cancer remain to be revealed.

In this study, we conducted a detailed bioinformatics analysis of the role of the FAM83 family by using the TCGA database in 33 cancers and identified correlations between FAM83 family genes and cancer by analyzing their expression, prognosis, tumor stemness, immune subtypes, tumor microenvironment, and drug sensitivity. We offer new evidence for the important role of FAM83 family genes in cancer, which may contribute to the discovery of new prognostic and therapeutic targets.

## Materials and methods

### TCGA data collection

We downloaded GDC TCGA pan-cancer data required for analysis from the UCSC Xena website (http://xena.ucsc.edu/) (Goldman et al., 2020), including RNA-Seq (HTSeq-FPKM), phenotype and survival data, immune subtypes, stemness indices according to mRNA expression (RNAss) and DNA methylation (DNAss). The TCGA pan-cancer data included 33 types with 11,057 samples, and the tumors and TCGA ID are shown in Table 1.

**TABLE 1:** TCGA pan-cancer data.

TCGA ID	Tumor types	Total samples	Tumor samples	Normal samples
ACC	Adrenocortical carcinoma	79	79	0
BLCA	Bladder urothelial carcinoma	430	411	19
BRCA	Breast invasive carcinoma	1,217	1,104	113
CESC	Cervical squamous cell carcinoma and endocervical adenocarcinoma	309	306	3
CHOL	Cholangiocarcinoma	45	36	9
COAD	Colon adenocarcinoma	512	471	41
DLBC	Lymphoid neoplasm diffuse large B-cell lymphoma	48	48	0
ESCA	Esophageal carcinoma	173	162	11
GBM	Glioblastoma multiforme	173	168	5
HNSC	Head and neck squamous cell carcinoma	546	502	44
KICH	Kidney chromophobe	89	65	24
KIRC	Kidney renal clear cell carcinoma	607	535	72
KIRP	Kidney renal papillary cell carcinoma	321	289	32
LAML	Acute myeloid leukemia	151	151	0
LGG	Brain lower grade glioma	529	529	0
LIHC	Liver hepatocellular carcinoma	424	374	50
LUAD	Lung adenocarcinoma	585	526	59
LUSC	Lung squamous cell carcinoma	550	501	49
MESO	Mesothelioma	86	86	0
OV	Ovarian serous cystadenocarcinoma	379	379	0
PAAD	Pancreatic adenocarcinoma	182	178	4
PCPG	Pheochromocytoma and paraganglioma	186	183	3
PRAD	Prostate adenocarcinoma	551	499	52
READ	Rectum adenocarcinoma	177	167	10
SARC	Sarcoma	265	263	2
SKCM	Skin cutaneous melanoma	472	471	1
STAD	Stomach adenocarcinoma	407	375	32
TGCT	Testicular germ cell tumors	156	156	0
THCA	Thyroid carcinoma	568	510	58
THYM	Thymoma	121	119	2
UCEC	Uterine corpus endometrial carcinoma	583	548	35
UCS	Uterine carcinosarcoma	56	56	0
UVM	Uveal melanoma	80	80	0
Total		11,057	10,327	730

### Pan-cancer analysis of FAM83 family expression

First, we plotted boxplots of FAM83 family expression across cancers, which can reflect the difference in gene expression dispersion. Then, differential expression analysis between cancer and paracancerous tissues was performed using the Wilcoxon signed-rank test. The heatmaps were created by the “pheatmap” R package based on the Log2 FC (log2-fold change) data. Among them, 15 cancer types with less than 5 paracancer samples were excluded. Next, Spearman correlation analysis was used to test the expression correlation between FAM83 family members. The Human Protein Atlas database contains the tissue and cellular distribution information of 24,000 human proteins (Uhlén et al., 2015; Yang et al., 2019). The database examines the distribution and expression of each protein in normal human tissues, tumor tissues, and cell lines by immunohistochemical techniques. The results are read by specialized personnel (Uhlén et al., 2015; Thul et al., 2017). We used this database to obtain an immunohistochemical expression profile of FAM83H across cancers.

### Pan-cancer analysis of FAM83 family survival

We divided the patients into high and low groups based on the median FAM83 family gene expression levels and plotted Kaplan-Meier (KM) plots to analyze prognosis between the two groups using the “survival and survminer” R package, with the log rank *p value*. *p value*s < 0.05 were considered to be statistically significant. Then, the prognostic value of FAM83 family members across cancers was further analyzed by Univariate Cox proportional hazard regression.

### Immune subtype analysis of the FAM83 family

Based on five representative immune signatures, such as monocytes or macrophages, wound healing, overall lymphocyte infiltration, IFN-g response, and TGF-b response (Thorsson et al., 2018), researchers have identified six immune subtypes in pan-cancer, including wound healing (C1), IFN-γ dominant (C2), inflammatory (C3), lymphocyte depleted (C4), immunologically quiet (C5), and TGF-β dominant (C6). Different distributions of immune subtypes exhibit differing clinical and biological characteristics, which to some extent determine the effects of tumor treatment (Thorsson et al., 2018). In pan-cancer, to understand the mRNA expression level of FAM83 family genes in six different immune subtypes of TCGA tumors, the differential expression was analyzed by the Kruskal-Wallis test.

### Stemness scores and the tumor microenvironment

The TME in antitumor therapy has important therapeutic and prognostic significance (Xiao and Yu, 2021). ESTIMATE is an algorithm that uses gene expression data to estimate stromal and immune cells in malignant tumor tissue (Yoshihara et al., 2013). The ESTIMATE algorithm is based on single sample Gene Set Enrichment Analysis (ssGSEA) and produces three scores, including StromalScore (which obtains the presence of stroma in cancer tissue), ImmuneScore (which stands for the infiltration of immune cells in cancer tissue), and ESTIMATEScore (which infers tumor purity) (Yoshihara et al., 2013). Thus, a Spearman correlation analysis was performed between the expression level of eight FAM83 genes and StromalScore and the tumor stemness score (RNAss and DNAss) by using the “estimate and limma” package.

### Drug sensitivity analysis

CellMiner is a web-based application that provides data on NCI-60 cancer cell lines and pharmacological profiles where we can mine these data (Shankavaram et al., 2009). The CellMiner website provides quick access to transcripts of 22,379 genes, 360 microRNAs and activity reports for 20,503 compounds (Reinhold et al., 2012). To explore the correlation between the transcript expression of FAM83 family genes and compound sensitivity, we downloaded FAM83 family gene transcript data and compound activity data from the CellMiner browser (https://discover.nci.nih.gov/cellminer/) (Reinhold et al., 2012). Raw data were preprocessed using the “impute” R package, and then, the Pearson correlation was used to test the correlation between gene expression and drug sensitivity. The drug responses of 262 FDA-approved or drugs on clinical trials were used in the correlation analysis.

### Statistical analysis

Wilcoxon signed-rank tests were used for differential expression analysis between cancer and paracancerous tissues. Log-rank tests or univariate Cox proportional hazard regression models were used to test the association between gene expression and patient overall survival. The Kruskal-Wallis test was used to test the association between gene expression and patient clinical characteristics and immune subtypes. Spearman’s or Pearson’s correlation was used to test the correlation between gene expression and stemness scores, StromalScore, ImmuneScore, ESTIMATEScore, and drug sensitivity. All statistical analyses were performed with R software (Version 3.5.3). Plots used in this manuscript were generated using the following R packages: ggpubr, pheatmap, ggplot2, survminer, and corrplot. All tests were two-tailed, and *p* values < 0.05 were considered statistically significant.

## Results

### Expression analysis of the FAM83 family in pan-cancer

**FIGURE 1:**
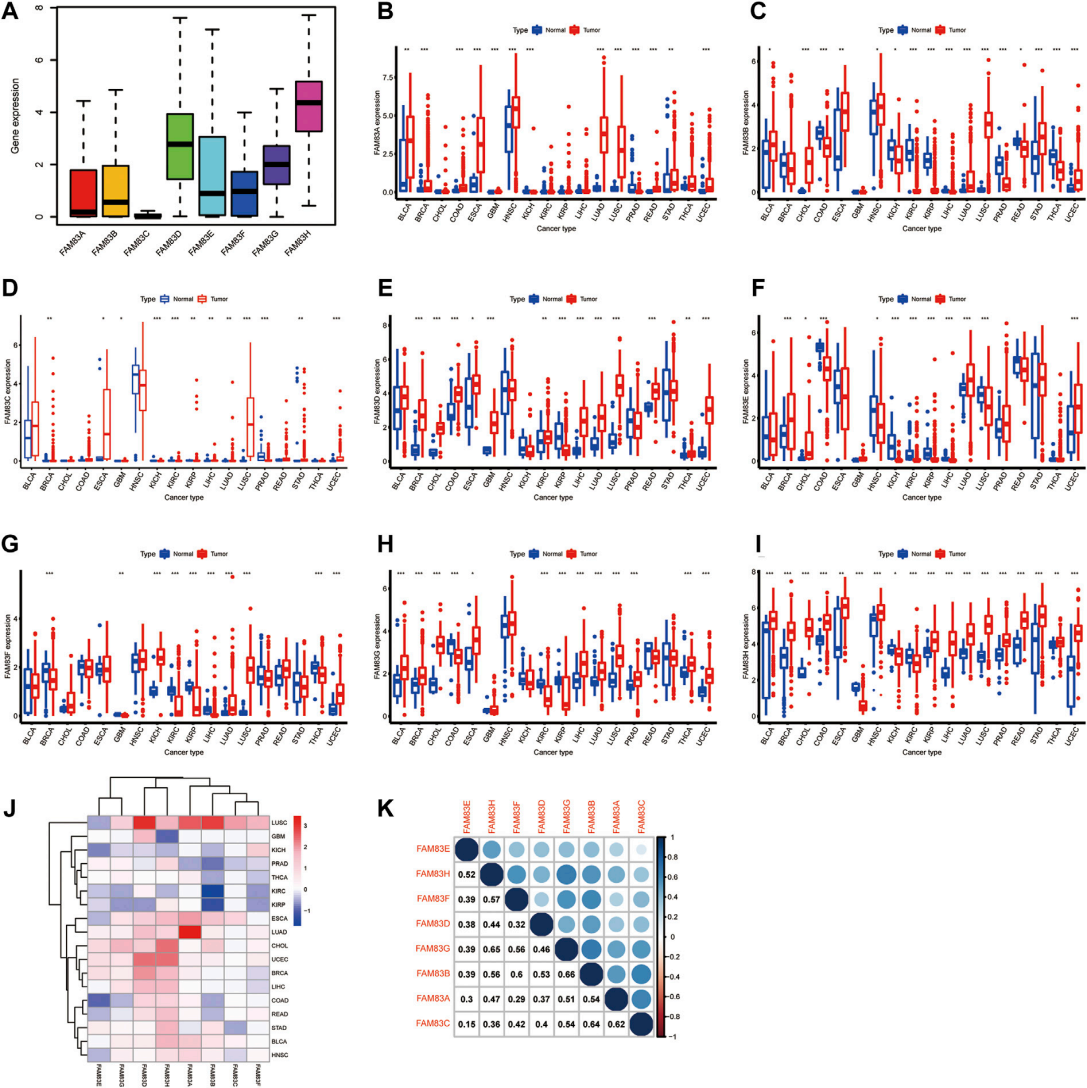
Analysis of the differential expression of FAM83 family genes across cancers. **(A)** Boxplot of the overall transcription level of the FAM83 family genes in 33 tumor types. **(B–I)** Boxplot of the difference in the expression of FAM83 family genes in carcinoma and normal paracancerous tissue (***p < 0.001; **p < 0.01; *p < 0.05). **(J)** Heatmap of FAM83 family gene expression in carcinoma and normal paracancerous tissues. Red stands for high expression, blue stands for low expression, and color depth indicates the degree of difference. **(K)** Relevance of expression among FAM83 family genes across cancers.

To understand the role of FAM83 family genes in cancer, we examined the expression levels of FAM83 family genes for all 33 tumor types in the TCGA database. The overall expression levels of eight members of the FAM83 gene family are shown (Figure 1A). Compared with FAM83D–H family members, FAM83A–C had lower average expression levels in all cancer types, with FAM83H having the highest expression. We performed an analysis of the expression of each member of the FAM83 family in tumors (Figures 1B–I). For the FAM83 family, we observed significant heterogeneity in the expression levels of different genes in the same tumor and the same gene in different tumors, with some high expression and some low expression. These findings suggest the importance of studying each gene as a whole.

**FIGURE 2:**
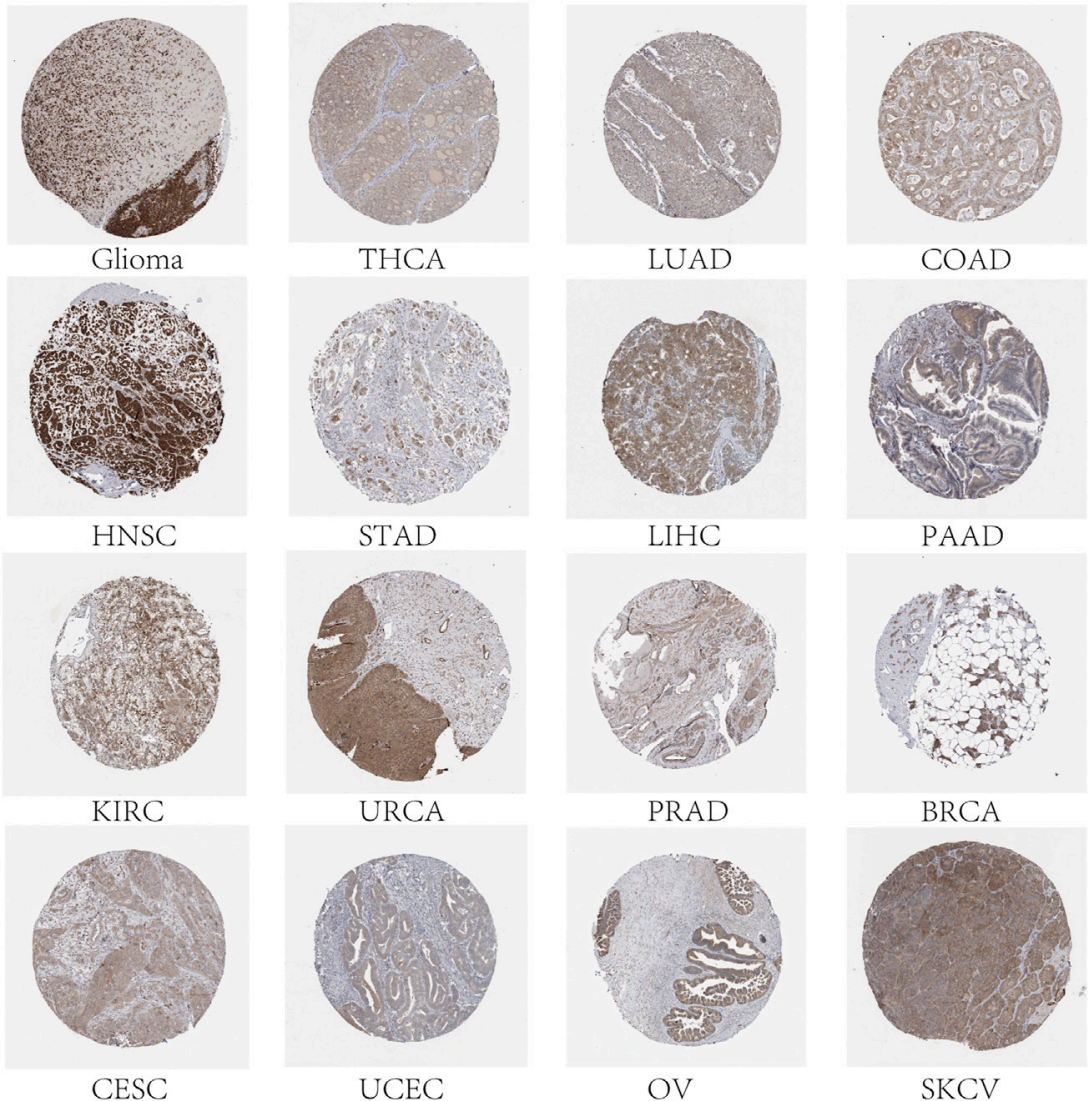
Expression level of the FAM83H protein. Immunohistochemical staining results showed expression of the FAM83H protein in 16 cancer types.

Characteristic of the role of genes in tumorigenesis is the dysregulation of gene expression, and there is growing evidence that the expression of FAM83 members is changed in a variety of tumor types. Most evidence is from studies conducted on animal or cellular models. Then, the Wilcoxon signed-rank test was used to analyze the differential expression of FAM83 family genes in cancer and paracancerous tissues (Figure 1J). The figure shows that the expression level of the FAM83 family fluctuates significantly in the dataset, which has excellent research significance. These eight FAM83 genes are either down- or upregulated in the majority of tumor types, and all eight members of the FAM83 family were upregulated in most tumors. For example, FAM83A was downregulated in PRAD, STAD, and KIRC, while it was highly expressed in other tumors. Obviously, LUAD, CHOL, UCEC, and BLCA were upregulated in all FAM83 family genes. Furthermore, expression levels of FAM83 genes were correlated positively with each other according to the Spearman correlation test, and we observed the highest correlation between FAM83B and FAM83G (*r* = 0.66), FAM83G and FAM83H (*r* = 0.65), FAM83B, and FAM83C (*r* = 0.64) (Figure 1K), suggesting that they may have some common roles or functions. In addition, immunohistochemical analysis of FAM83H protein expression in 16 cancers was performed based on the “Human Protein Atlas” database of representative protein expression (Figure 2).

### Clinical correlation analysis

**FIGURE 3:**
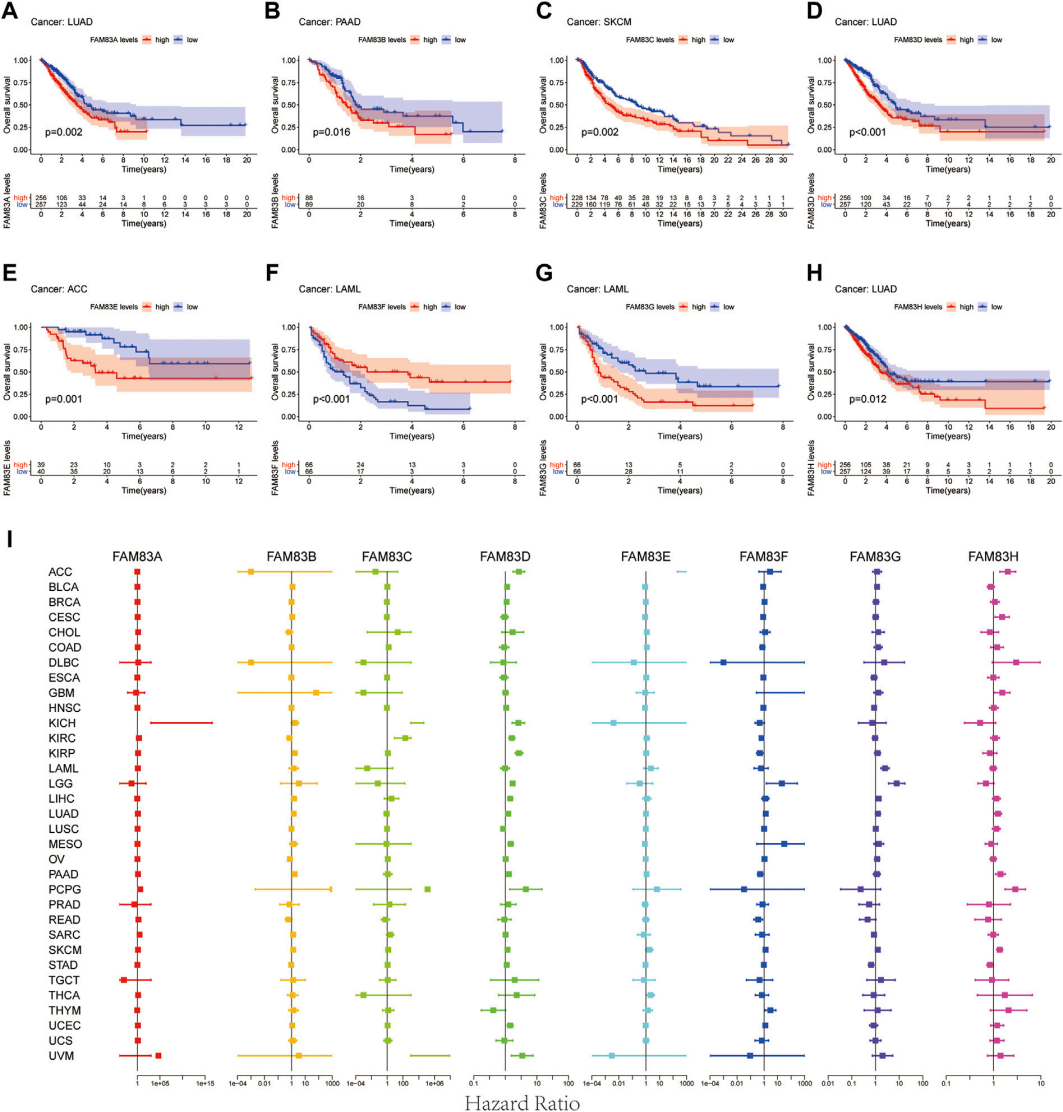
Survival analysis of FAM83 family genes across cancers. **(A–H)** KM curves for the prognostic value of the FAM83 family gene. Only partial survival charts with *p* < 0.05 are displayed. **(I)** Univariate Cox proportional hazard regression analysis between the FAM83 family and overall survival. A hazard ratio >1 is considered a high-risk factor for cancer, with an adverse prognostic outcome. In contrast, a hazard ratio of <1 is deemed a low-risk factor for cancer.

**FIGURE 4:**
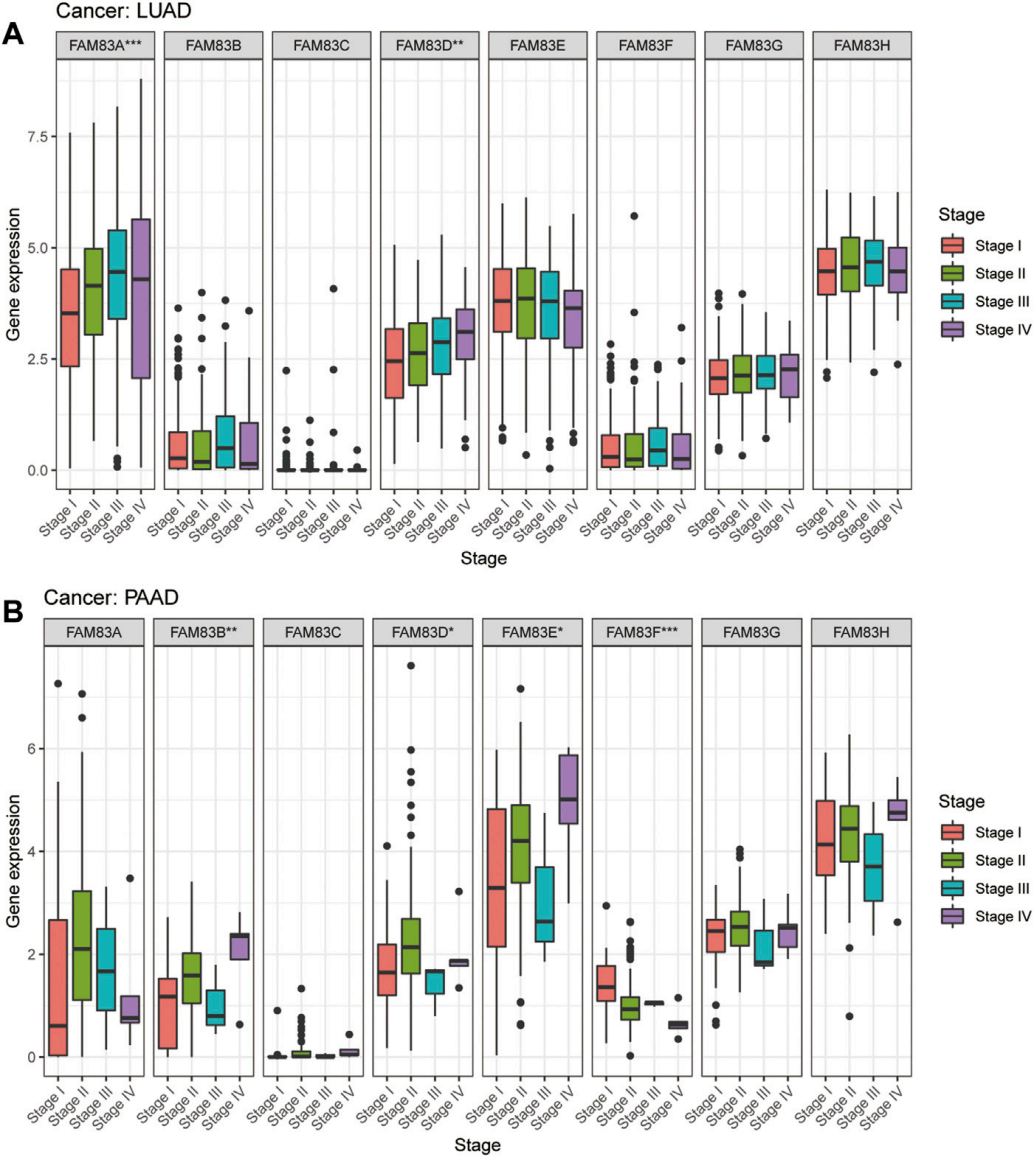
Expression analysis of FAM83 family genes in cancer staging. **(A)** LUAD. **(B)** PAAD. (***p < 0.001; **p < 0.01; *p < 0.05).

To predict which FAM83 member promotes or suppresses tumorigenesis in which cancer type, we performed Kaplan-Meier analysis of FAM83 family genes in 33 TCGA tumors. Survival time and survival status of patients were obtained from the UCSC Xena website (Supplementary Table S1). The partial survival diagram is shown in Figure 3 (the analysis results are shown in Supplementary Table S2). We found that altered levels of FAM83 gene expression were usually related to patient prognosis. High expression of FAM83A was notably correlated with a worse prognosis in LUAD (*p* = 0.002, Figure 3A), KIRP, KIRC, PAAD, SKCM, UCEC, and LUSC (Supplementary Table S2). Comparatively, low expression of FAM83B was linked to a poorer prognosis for CHOL and READ, while high expression of FAM83B resulted in worse survival for PAAD (*p* = 0.016, Figure 3B) and MESO (Supplementary Table S2). Low expression of FAM83C was correlated with a good prognosis of SKCM (*p* = 0.002, Figure 3C). Similarly, underexpression of FAM83D and FAM83E may be a good signal for prognosis in patients with ACC (*p* = 0.001, Figure 3D) and LUAD (*p* < 0.001, Figure 3E). High FAM83F expression was correlated with a better prognosis in LAML (*p* < 0.001, Figure 3F). In contrast, poor expression of FAM83G may be a beneficial signal for LAML prognosis (*p* < 0.001, Figure 3G). Meanwhile, low FAM83H expression was associated with a better prognosis in LUAD patients (*p* = 0.012, Figure 3H). In addition, univariate Cox proportional hazard regression analysis was used to demonstrate the association between FAM83 family gene pan-cancer expression and patient overall survival rate (Figure 3I). In addition, we analyzed the connection between staging and FAM83 gene expression levels. Distribution of staging across cancers is shown in Supplementary Table S3. In LUAD, the expression of FAM83A and FAM83D was found to be associated with TNM stage. Both had low expression levels in stage I and high levels in stages II, III, and IV. In PAAD, the expression of FAM83B, FAM83D, FAM83E, and FAM83F was found to be associated with TNM stage (Figure 4A). The expression levels of FAM83B and FAM83D were highest in stage IV, and conversely, the expression of FAM83F was lowest in stage IV (Figure 4B). Therefore, the difference in FAM83 family expression in different TNM stages can be used as a predictor to predict cancer progression in clinical treatment.

### Analysis of immune subtype correlations in FAM83 family genes

**FIGURE 5:**
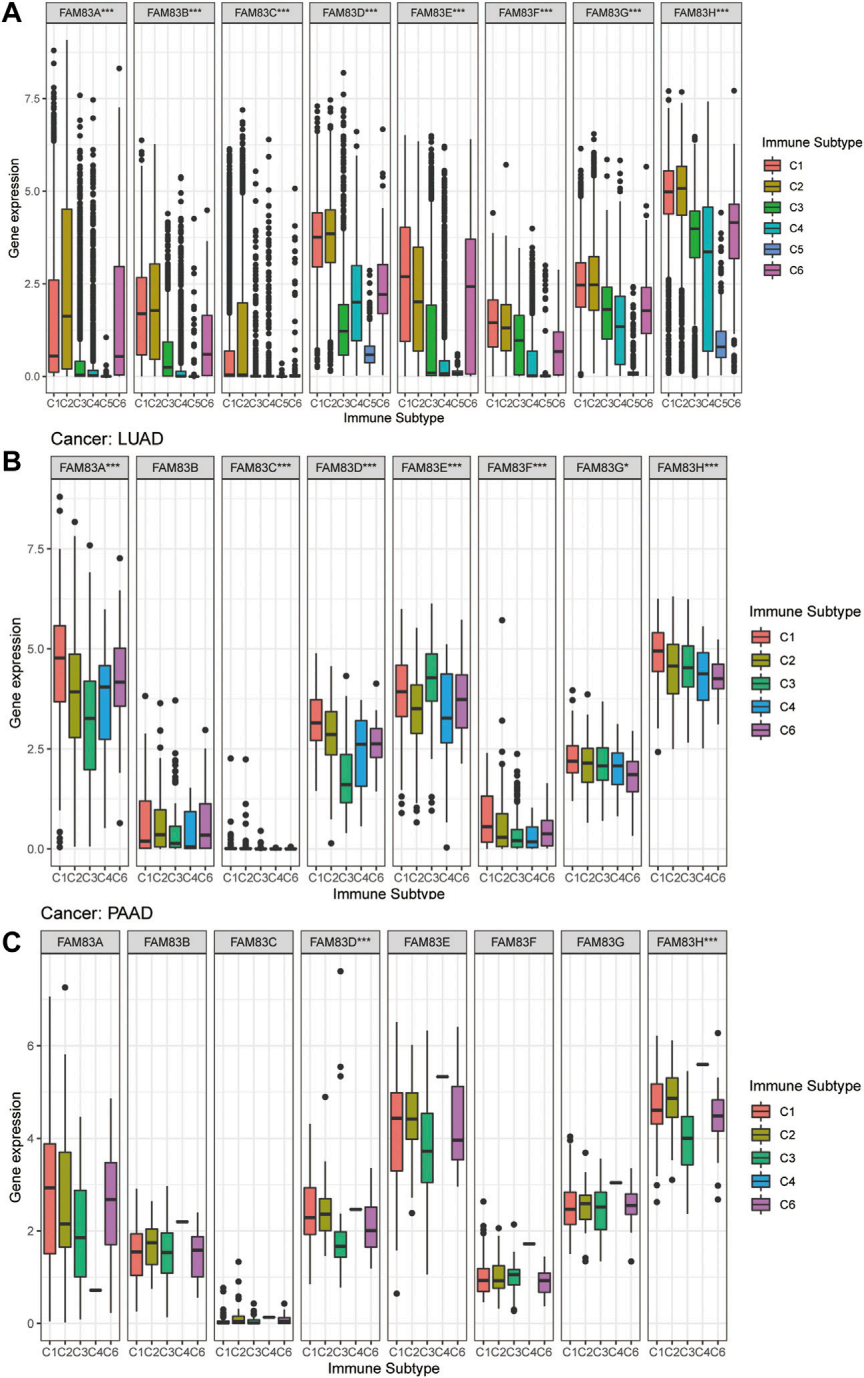
Expression analysis of FAM83 family genes in different immune subtypes. **(A)** Boxplots of the overall transcript levels of FAM83 family genes in the C1–C6 immune subtypes across cancers. **(B)** LUAD. **(C)** PAAD. (***p < 0.001; **p < 0.01; *p < 0.05).

To understand the expression level of FAM83 family members in six different immune subtypes of TCGA tumors, the differential expression was analyzed using the Kruskal-Wallis test, and distribution of immune subtypes across cancers is shown in Supplementary Table S4. We observed that the expression of FAM83 family genes in the six different immune subtypes was significantly different, and the *p* values were all less than 0.001 (Figure 5A), indicating that the FAM83 family might be related to tumor immunity. Obviously, FAM83H ranks first in the global expression level of C1-C6 (Figure 5A). Moreover, the expression of the FAM83 family in immune subtypes is also different among different tumor types. Among the immune subtypes of LUAD, all members were differentially expressed except FAM83B. Among them, FAM83A expression was highest in C1, while FAM83A expression was lowest in C3 (Figure 5B). In PAAD, only FAM83D and FAM83H were significantly differentially expressed in the six immune subtypes (Figure 5C).

### Stemness scores and the tumor microenvironment

**FIGURE 6:**
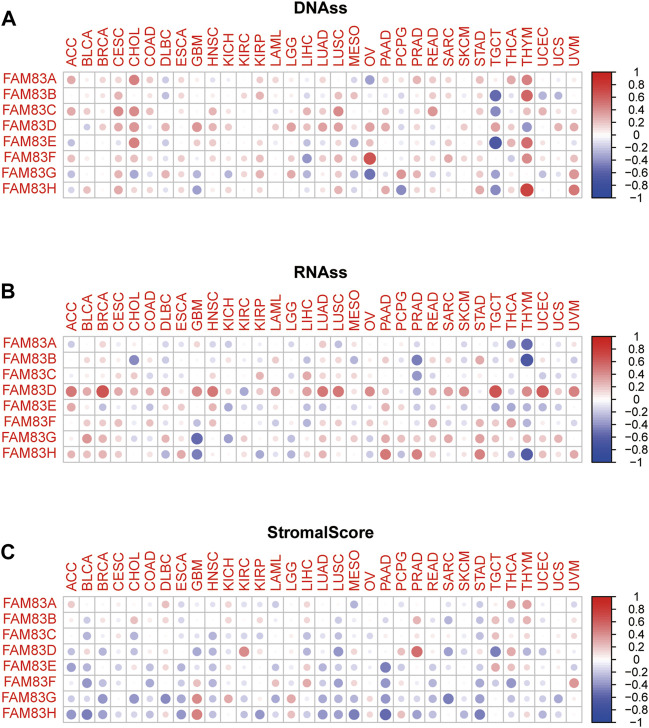
Relationship of FAM83 family gene expression levels with tumor microenvironment and stemness scores across cancers. **(A,B)** Heatmap of the correlation between FAM83 family genes and RNAss and DNAss. **(C)** Heatmap of the correlation between FAM83 family genes and StromalScore. The circle color indicates the correlation coefficient, red indicates a positive correlation, and blue indicates a negative correlation.

**FIGURE 7:**
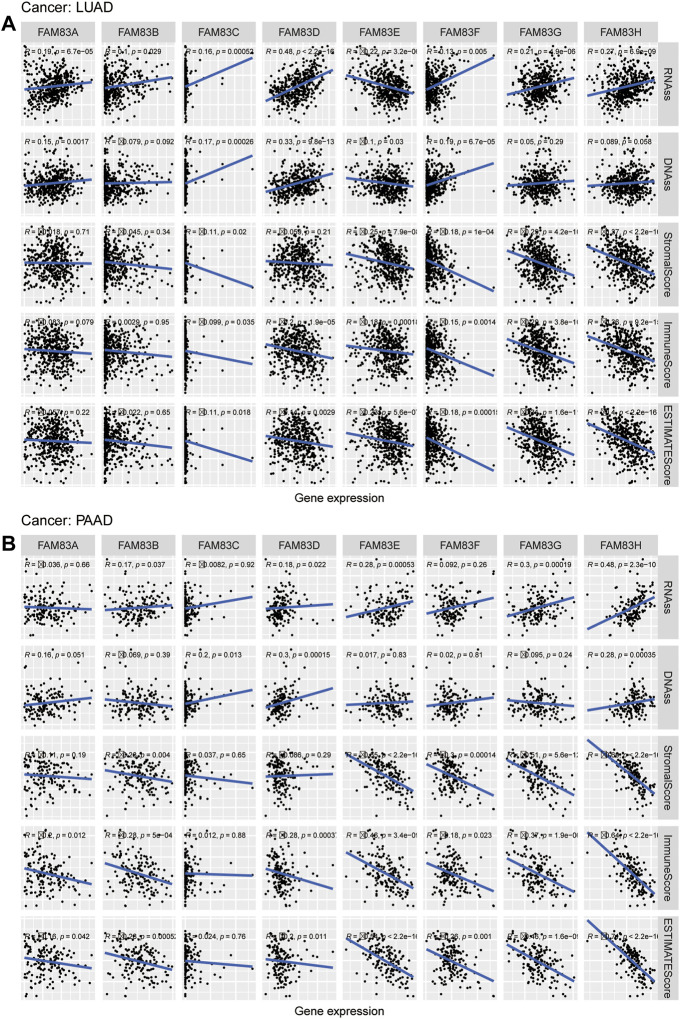
Relevance analysis of the FAM83 family genes with stemness scores and microenvironment scores in LUAD **(A)** and PAAD **(B)**.

The stromal score of TCGA tumor samples was calculated by applying the ESTIMATE algorithm (Yoshihara et al., 2013). Spearman correlation analysis was conducted between the expression level of FAM83 family genes and RNAss, DNAss, and Stromalscore (Figure 6). FAM83 family genes showed varying degrees of correlation with RNAss and DNAss across cancers. Apparently, there was a significant association between THYM and FAM83 family genes in DNAss, with positive correlations with FAM83A (*r* = 0.45, *p* < 0.001), FAM83B (*r* = 0.57, *p* = 0), FAM83C (*r* = 0.23, *p* = 0.01), FAM83E (*r* = 0.54, *p* < 0.001), FAM83F (*r* = 0.42, *p* < 0.001), and FAM83H (*r* = 0.74, *p* = 0) and negative correlations with FAM83D (*r* = −0.39, *p* < 0.001) (Figure 6A). In RNAss, there was a remarkable positive relationship between RNAss and FAM83D in many tumors (Figure 6B). In addition, we found a negative correlation between the FAM83 family genes and StromalScore in most tumors (Figure 6C). For example, the expression of FAM83H was negatively associated with PAAD, MESO, BLCA, STAD, ACC, LUAD, ESCA, KIRP, PRAD, BRCA, and LUSC. However, FAM83A is positively related to the THYM StromalScore (*r* = 0.33, *p* < 0.001). Furthermore, we analyzed the relevance of the FAM83 family genes to ImmuneScore, Tumor Purity and ESTIMATEScore (Supplementary Figure S1) using the ESTIMATE method to infer the ratio of stromal and immune cells in malignant tumors and thus tumor purity in malignant tumors (Yoshihara et al., 2013; Becht et al., 2016). In LUAD and PAAD, the FAM83 family has different correlations with RNAss, DNAss, StromalScore, ImmuneScore, and ESTIMATEScore. Moreover, most of the FAM83 family members showed a negative correlation with the StromalScore, ImmuneScore, and ESTIMATEScore, indicating that FAM83 family gene expression is associated with higher tumor purity (Figure 7).

### Correlation analysis of drug sensitivity

**FIGURE 8:**
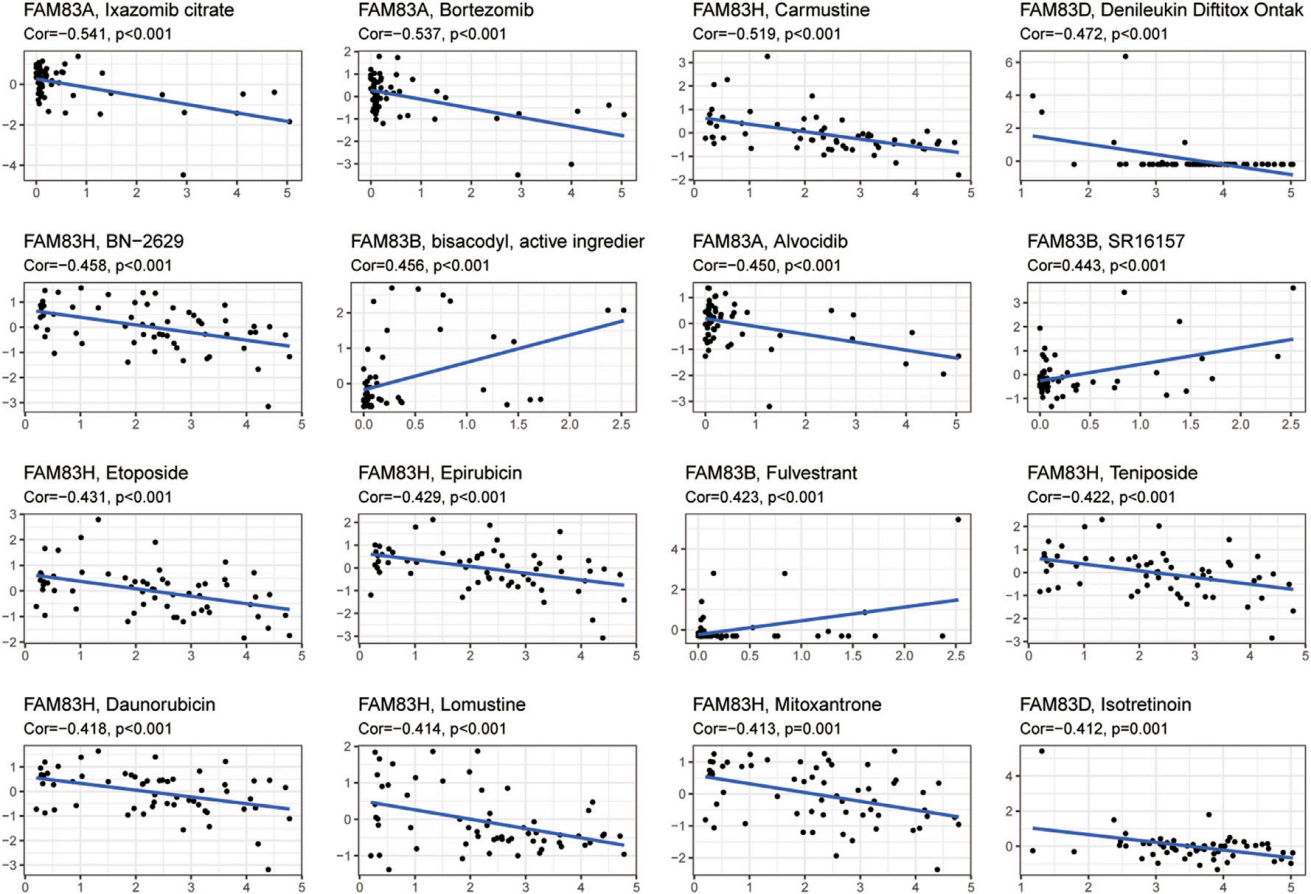
Correlation analysis between FAM83 family gene expression and drug sensitivity. Partial scatter plots are shown, and the scatter plots are sorted by *p value* by *p* < 0.05 choice.

We next investigated the correlation between FAM83 family gene expression and compound activity (Supplementary Table S5). We discovered that high expression of the FAM83 family genes, particularly FAM83A and FAM83H, was associated with increased resistance to many antitumor drugs (Figure 8). For example, FAM83A was positively correlated with drug resistance to ixazomib citrate, bortezomib, and alvocidib, and FAM83H was associated with drug resistance to carmustine, etoposide, epirubicin, teniposide, daunorubicin, lomustine, mitoxantrone, and BN-2629. We also observed that some genes were related to drug sensitivity. For example, FAM83B was related to the drug sensitivity of diazoxide, SR16157, and fulvestrant.

## Discussion

Dysregulation of the expression levels of the FAM83 family genes plays a vital role in tumor development, including tumor proliferation, metastasis, tumor resistance, and tumor biomarkers (Liu et al., 2020; Rong et al., 2020; He et al., 2021). In previous studies, the FAM83 family was shown to regulate tumor progression and promote tumor chemoresistance through its association with various signaling pathways. Initially, the FAM83 family of oncogenes was first identified by the Bissell and Jackson laboratories using two new methods (Cipriano et al., 2012; Lee et al., 2012). They found that FAM83A and FAM83B are candidate cancer-related genes that can develop FAM83 resistance to EGFR-TKIs. These data provide important insights into drug resistance to TKIs (Lee et al., 2012). Subsequently, there have been an increasing number of discussions about the role of FAM83 family genes in tumors, but the role of FAM83 family genes across cancers has not been thoroughly explored.

Here, we used a bioinformatics approach to analyze the biological properties of the FAM83 family genes in pan-cancer. We found remarkable heterogeneity in the expression levels of the FAM83 family genes in tumors and performed a series of clinical correlation analyses. FAM83A expression was found to be significantly upregulated in tumors such as lung, breast, and head and neck squamous cell carcinomas and had a poor prognosis in cancer. Recent studies have also found that when FAM83A is knocked down, it increases trastuzumab sensitivity in drug-resistant cells and may be a target for the development of clinical diagnostic or therapeutic strategies to guide the treatment of patients with HER2+ breast cancer who have developed trastuzumab resistance (Boyer et al., 2013). Moreover, activation of the Wnt/β-catenin signaling pathway by FAM83A promotes the growth and metastasis of HNSC cells (Ji et al., 2021). Furthermore, Wang et al. (2019b) found that circ-ZKSCAN1 could reduce mir-330-5p activity and promote FAM83A expression, leading to inhibition of the MAPK signaling pathway and thus promoting NSCLC progression. In LUAD, the expression of FAM83A was associated with TNM stage with low expression levels in stage I and high levels in stages II, III, and IV. It may serve as a good biomarker. In addition, the expression level of FAM83A in the TME was negatively related to the stromal score of lung cancer and pancreatic cancer, indicating that high expression of FAM83A was correlated with higher tumor purity. FAM83A was positively correlated with resistance to gefitinib, bosutinib, imatinib, lapatinib and erlotinib (Additional file 1). All these findings are consistent with the literature research.

Our analysis showed that FAM83B was notably overexpressed in lung cancer, gastric cancer, and endometrial cancer, and Cipriano et al. previously found that FAM83B promotes the proliferation and deterioration of tumor-derived cells or RAS-transformed mammary epithelial cells (Cipriano et al., 2012). Subsequent studies demonstrated that FAM83A and FAM83B are biomarkers of lung cancer prognosis and potential new treatment targets (Okabe et al., 2015; Richtmann et al., 2019). In addition, studies have shown that FAM83B inhibits endometrial cancer cell migration and invasion by silencing the PI3K/AKT pathway (Lin et al., 2019). In addition, the high expression of FAM83B in PAAD suggests a poor prognosis and is significantly associated with the TNM stage, with stage IV expression being significantly higher than that of stages I, II, and III, which is in line with the conclusion of Shen et al. (2017). Importantly, we found that FAM83B was negatively correlated with cisplatin and carboplatin resistance, as well as with the ovarian cancer tumor microenvironment in tumor purity, and related studies also showed that FAM83B suppresses cisplatin resistance and slows ovarian tumor progression (He et al., 2021), indicating that the biological properties of the FAM83 family are not consistent in different tumors.

FAM83D upregulation is recognized to have a critical role in the cell division and deterioration of many tumors, and our study shows that FAM83D is upregulated in almost all cancers except KIRP and suggests a poor prognosis. Silencing of FAM83D inhibits glioblastoma progression and increases sensitivity to temozolomide (Wang et al., 2022). Studies have suggested that knockdown of FAM83D inhibits the growth of LUAD cells by downregulating cell cycle arrest, and it may emerge as a novel therapeutic target, prognostic marker and prognostic marker for LUAD (Shi et al., 2016). However, we revealed that FAM83D was highly expressed in LUSC and positively associated with tumor purity, but KM survival analysis indicated a better prognosis. This warrants further study at the cellular and animal levels. FAM83H was originally identified as a protein essential for tooth enamel formation, and its function in tooth development is well known to humans (Kim et al., 2008). However, in recent years, a role in tumor development has been proposed, and we found that FAM83H expression was upregulated in most tumors. A study found that FAM83H and SCRIB synergistically activate gastric cancer progression by stabilizing β-catenin (Hussein et al., 2020). However, our analysis showed that FAM83H had a better prognosis in the survival analysis of gastric cancer, which is not consistent with the above results. This may stem from the different data of the study, and further support from more data is needed. Additionally, studies on FAM83C, FAM83E, FAM83F, and FAM83G in tumors are still lacking. Recently, overexpression of FAM83C and FAM83E was shown to promote the transformation of human mammary epithelial cells (Cipriano et al., 2014). Liu et al. found that FAM83G could act as an oncogenic factor to predict overall survival in patients with hepatocellular carcinoma and facilitate the progression of hepatocellular carcinoma cells by activating the PI3K/AKT signaling pathway (Liu et al., 2021). Our study also presents a good systematic analysis of the expression and prognosis of these genes.

Most tumors lack validated prognostic factors, so the search for new biomarkers can be of great benefit to cancer treatment. The FAM83 family is a family of oncogenes worthy of study and is widely involved in human cancer. The FAM83 family is significantly correlated with the prognosis of various cancers, revealing the significance of FAM83 family genes in guiding the prognosis of cancer patients. However, the expression of FAM83 family genes is low in some tumors, and this unusual situation needs to be further investigated to deepen our understanding of the role of FAM83 family proteins in cancer development.

Subsequently, the C1–C6 immune subtype discovered by Thorsson et al. (2018) is present in all human malignancies, forming a specific immune environment with an essential role in cancer prognosis and predicting disease outcomes. Intriguingly, we observed that all FAM83 family genes were related to the immune infiltrative subtype in the TME, and FAM83 family genes were highly expressed in C1, C2, and C6, indicating a worse prognosis. The interaction between the TME and tumor cells plays a decisive role in tumorigenesis, progression and treatment. The tumor microenvironment as a therapeutic target for cancer has attracted extensive research and clinical interest (Xiao and Yu, 2021). Using the StromalScore and ImmuneScore, we analyzed the correlation between the expression levels of FAM83 family genes and tumor purity and tumor microenvironment characteristics. It was clear that the FAM83 family is positively correlated with tumor purity in the majority of cancers.

A study showed that cancer stem cells function similarly to stem cell self-renewal (Friedmann-Morvinski and Verma, 2014). Defining tumor stemness by new metrics provides a comprehensive dedifferentiation profile as a new and significant hallmark of cancer (Malta et al., 2018). We confirmed the role of the FAM83 family genes in tumor stem cell characterization by analyzing the association between FAM83 family genes and RNAss and DNAss. Finally, we investigated the drug sensitivity of the FAM83 family genes. We found that elevated expression levels of FAM83 family genes were associated with increased resistance to many chemotherapeutic agents, particularly FAM83A and FAM83H. For example, FAM83A was positively correlated with drug resistance to ixazomib citrate, bortezomib, alvocidib, and FAM83H and was associated with drug resistance to carmustine, etoposide, epirubicin, teniposide, daunorubicin, lomustine, mitoxantrone, and BN-2629. We also noted that some genes are linked to drug sensitivity. For instance, FAM83B is associated with drug sensitivity to bisacodyl, SR16157, and fulvestrant. This shows that FAM83 family genes play an important role in tumor resistance or sensitivity. This may offer new therapeutic options for the effectiveness of oncology chemotherapy and identify new molecular biomarkers for predicting chemotherapy resistance.

In conclusion, we provide a full and detailed overview of the role of the FAM83 family genes in expression, survival and the tumor microenvironment, which has enormous value as novel clinical diagnostic and therapeutic targets. Of course, our study also has limitations. First, the results of this study have not been validated by other independent databases, so our future work is to validate it by our data and other public databases. More importantly, the data of this study are based on bioinformatics analysis and lack the results of *in vitro* and *in vivo* experiments. Therefore, we plan to further reveal the role of FAM83 family genes in tumors through molecular and animal experiments.

## Conclusion

In this study, we analyzed the expression and prognostic value of FAM83 family members across cancers. In addition, we learned that the molecular biology of cancer is highly heterogeneous, providing new clues for cancer diagnosis and treatment. Our findings also suggest that FAM83A/D/G/H is significantly upregulated in the majority of tumor types and correlates with poor patient prognosis. Furthermore, FAM83 family genes are significantly associated with immune subtypes, suggesting that the FAM83 family may be associated with tumor immunity. Additionally, we analyzed the correlation between FAM83 family genes and tumor purity, which can be inferred from the tumor purity in the tumor microenvironment. Moreover, the relationship between the FAM83 family and drug sensitivity was analyzed. In summary, our study will largely help to reveal their role in tumorigenesis. This is crucial for the development of personalized drugs for cancer therapy. These findings provide novel evidence for future studies on the role of FAM83 family members in cancer development and treatment.

## Data Availability

The original contributions presented in the study are included in the article/Supplementary Material, further inquiries can be directed to the corresponding author.
