# Supplementary material for: Pan-cancer analysis of the FAM83 family and its association with prognosis and tumor microenvironment
Source: Front Genet. 2022 Jul 22;13:919559. doi: 10.3389/fgene.2022.919559 (PMC9353330; doi:10.3389/fgene.2022.919559)
Supplement: Supplementary file 1 [file Presentation2.PPT]

## Slide 1
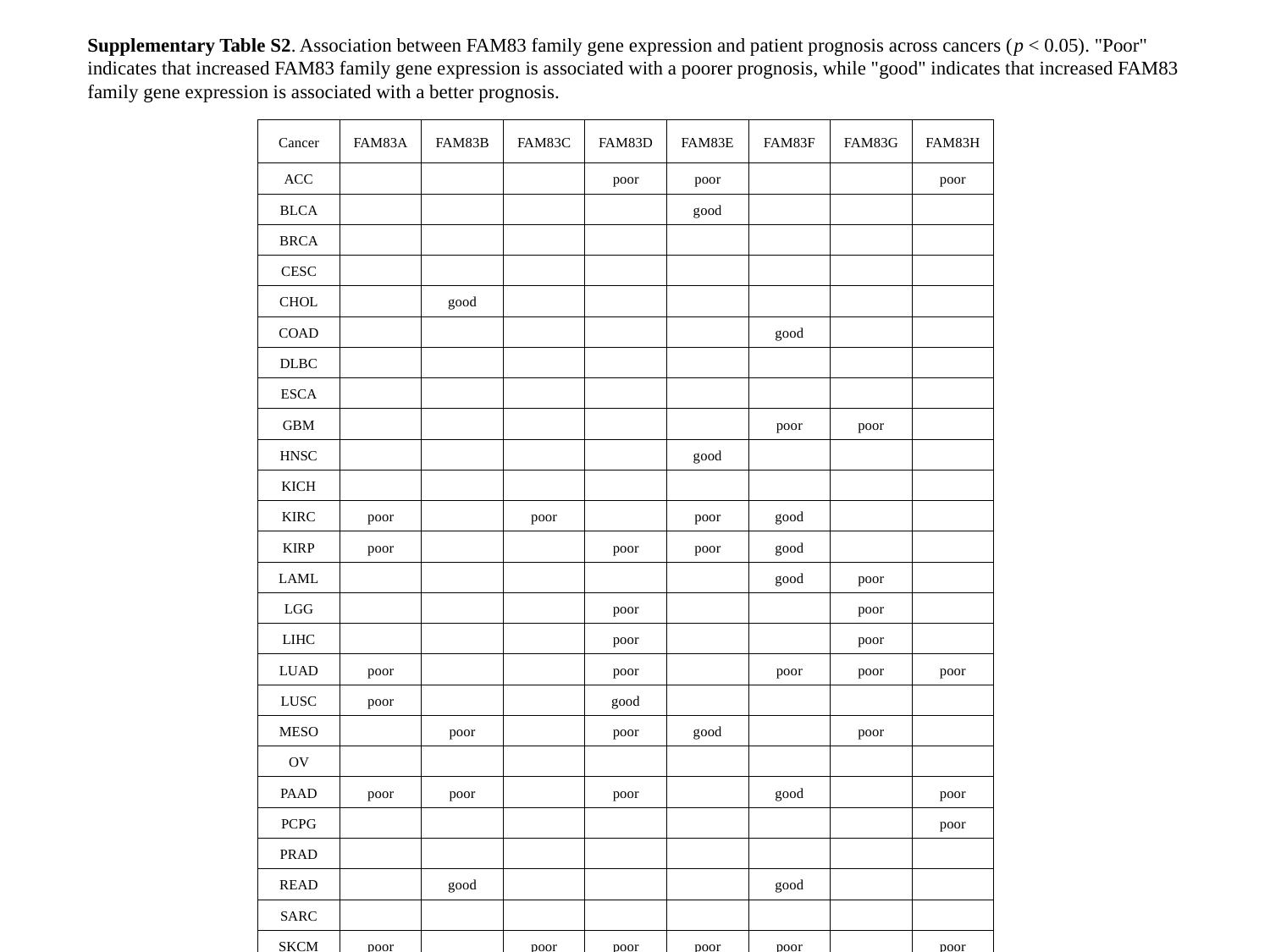

# Supplementary Table S2. Association between FAM83 family gene expression and patient prognosis across cancers (p < 0.05). "Poor" indicates that increased FAM83 family gene expression is associated with a poorer prognosis, while "good" indicates that increased FAM83 family gene expression is associated with a better prognosis.
| Cancer | FAM83A | FAM83B | FAM83C | FAM83D | FAM83E | FAM83F | FAM83G | FAM83H |
| --- | --- | --- | --- | --- | --- | --- | --- | --- |
| ACC | | | | poor | poor | | | poor |
| BLCA | | | | | good | | | |
| BRCA | | | | | | | | |
| CESC | | | | | | | | |
| CHOL | | good | | | | | | |
| COAD | | | | | | good | | |
| DLBC | | | | | | | | |
| ESCA | | | | | | | | |
| GBM | | | | | | poor | poor | |
| HNSC | | | | | good | | | |
| KICH | | | | | | | | |
| KIRC | poor | | poor | | poor | good | | |
| KIRP | poor | | | poor | poor | good | | |
| LAML | | | | | | good | poor | |
| LGG | | | | poor | | | poor | |
| LIHC | | | | poor | | | poor | |
| LUAD | poor | | | poor | | poor | poor | poor |
| LUSC | poor | | | good | | | | |
| MESO | | poor | | poor | good | | poor | |
| OV | | | | | | | | |
| PAAD | poor | poor | | poor | | good | | poor |
| PCPG | | | | | | | | poor |
| PRAD | | | | | | | | |
| READ | | good | | | | good | | |
| SARC | | | | | | | | |
| SKCM | poor | | poor | poor | poor | poor | | poor |
| STAD | | | | | | | | good |
| TGCT | good | | | | | | | |
| THCA | | | | | | | | |
| THYM | | | | | | | | poor |
| UCEC | poor | | | poor | | | | poor |
| UCS | | | | | | | | |
| UVM | | | poor | | | | poor | |

## Slide 2
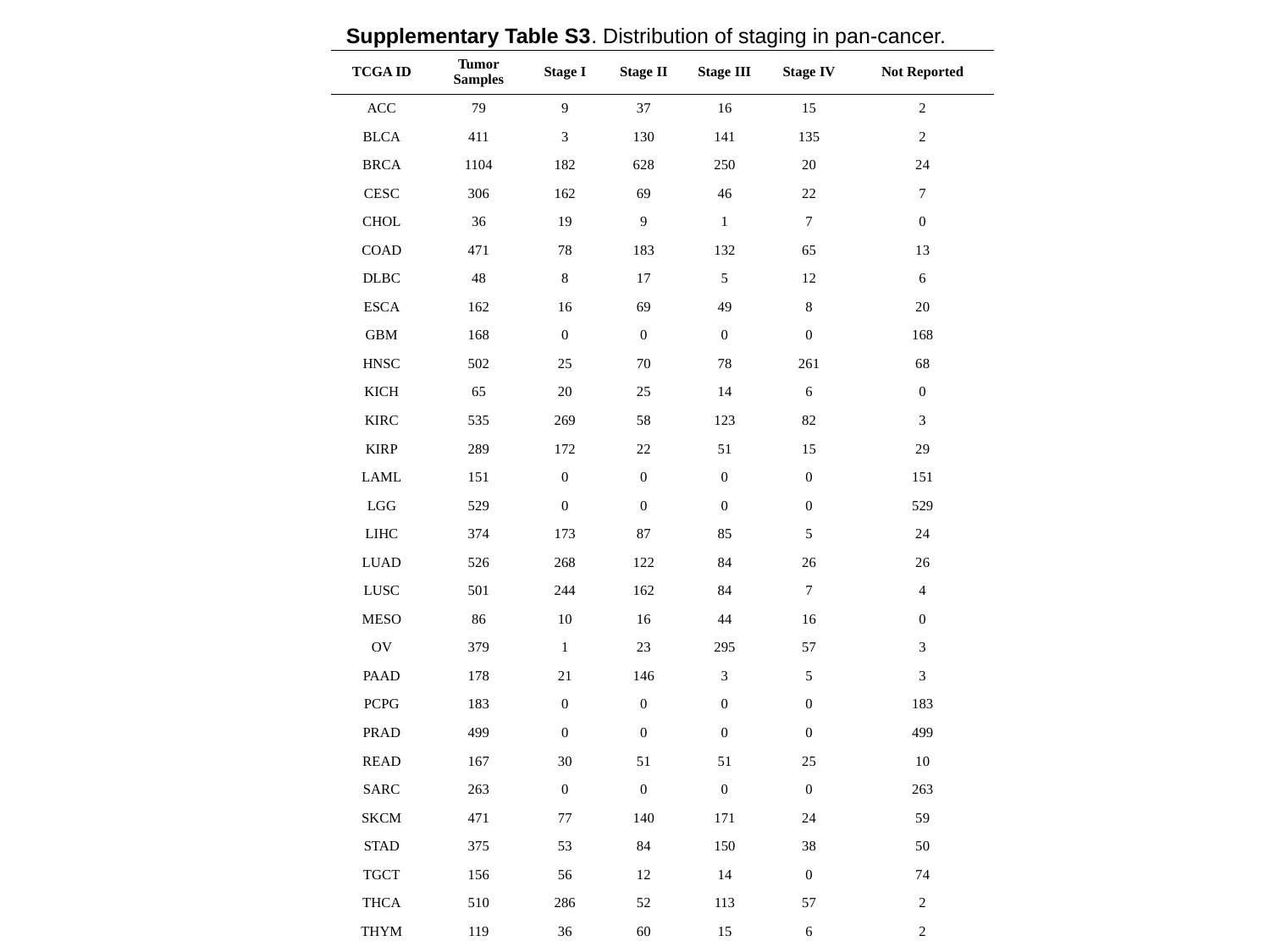

# Supplementary Table S3. Distribution of staging in pan-cancer.
| TCGA ID | Tumor Samples | Stage I | Stage II | Stage III | Stage IV | Not Reported |
| --- | --- | --- | --- | --- | --- | --- |
| ACC | 79 | 9 | 37 | 16 | 15 | 2 |
| BLCA | 411 | 3 | 130 | 141 | 135 | 2 |
| BRCA | 1104 | 182 | 628 | 250 | 20 | 24 |
| CESC | 306 | 162 | 69 | 46 | 22 | 7 |
| CHOL | 36 | 19 | 9 | 1 | 7 | 0 |
| COAD | 471 | 78 | 183 | 132 | 65 | 13 |
| DLBC | 48 | 8 | 17 | 5 | 12 | 6 |
| ESCA | 162 | 16 | 69 | 49 | 8 | 20 |
| GBM | 168 | 0 | 0 | 0 | 0 | 168 |
| HNSC | 502 | 25 | 70 | 78 | 261 | 68 |
| KICH | 65 | 20 | 25 | 14 | 6 | 0 |
| KIRC | 535 | 269 | 58 | 123 | 82 | 3 |
| KIRP | 289 | 172 | 22 | 51 | 15 | 29 |
| LAML | 151 | 0 | 0 | 0 | 0 | 151 |
| LGG | 529 | 0 | 0 | 0 | 0 | 529 |
| LIHC | 374 | 173 | 87 | 85 | 5 | 24 |
| LUAD | 526 | 268 | 122 | 84 | 26 | 26 |
| LUSC | 501 | 244 | 162 | 84 | 7 | 4 |
| MESO | 86 | 10 | 16 | 44 | 16 | 0 |
| OV | 379 | 1 | 23 | 295 | 57 | 3 |
| PAAD | 178 | 21 | 146 | 3 | 5 | 3 |
| PCPG | 183 | 0 | 0 | 0 | 0 | 183 |
| PRAD | 499 | 0 | 0 | 0 | 0 | 499 |
| READ | 167 | 30 | 51 | 51 | 25 | 10 |
| SARC | 263 | 0 | 0 | 0 | 0 | 263 |
| SKCM | 471 | 77 | 140 | 171 | 24 | 59 |
| STAD | 375 | 53 | 84 | 150 | 38 | 50 |
| TGCT | 156 | 56 | 12 | 14 | 0 | 74 |
| THCA | 510 | 286 | 52 | 113 | 57 | 2 |
| THYM | 119 | 36 | 60 | 15 | 6 | 2 |
| UCEC | 548 | 341 | 51 | 127 | 29 | 0 |
| UCS | 56 | 21 | 5 | 20 | 10 | 0 |
| UVM | 80 | 0 | 39 | 36 | 4 | 1 |
| Total | 10327 | 2580 | 2367 | 2198 | 957 | 2225 |

## Slide 3
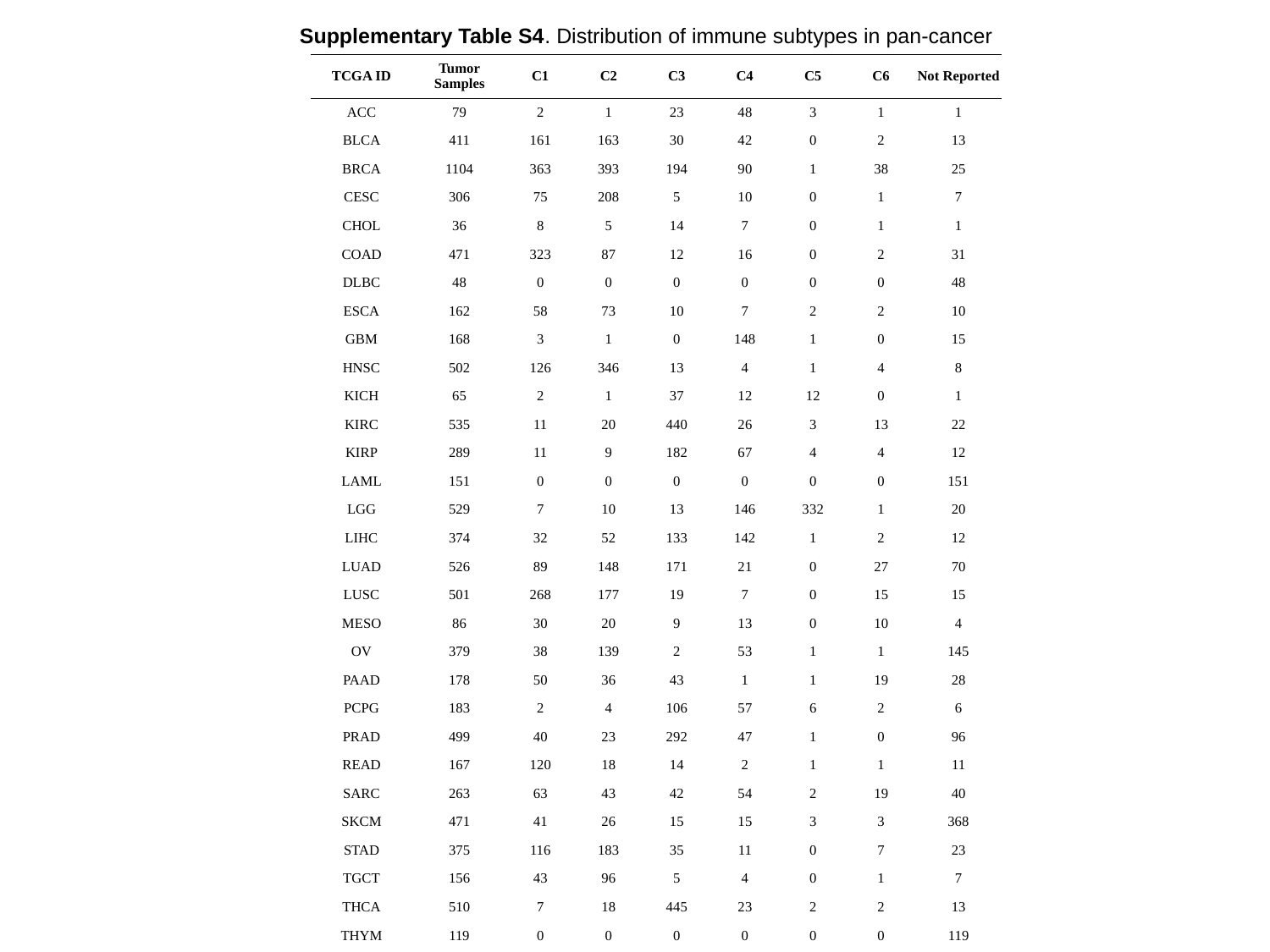

# Supplementary Table S4. Distribution of immune subtypes in pan-cancer
| TCGA ID | Tumor Samples | C1 | C2 | C3 | C4 | C5 | C6 | Not Reported |
| --- | --- | --- | --- | --- | --- | --- | --- | --- |
| ACC | 79 | 2 | 1 | 23 | 48 | 3 | 1 | 1 |
| BLCA | 411 | 161 | 163 | 30 | 42 | 0 | 2 | 13 |
| BRCA | 1104 | 363 | 393 | 194 | 90 | 1 | 38 | 25 |
| CESC | 306 | 75 | 208 | 5 | 10 | 0 | 1 | 7 |
| CHOL | 36 | 8 | 5 | 14 | 7 | 0 | 1 | 1 |
| COAD | 471 | 323 | 87 | 12 | 16 | 0 | 2 | 31 |
| DLBC | 48 | 0 | 0 | 0 | 0 | 0 | 0 | 48 |
| ESCA | 162 | 58 | 73 | 10 | 7 | 2 | 2 | 10 |
| GBM | 168 | 3 | 1 | 0 | 148 | 1 | 0 | 15 |
| HNSC | 502 | 126 | 346 | 13 | 4 | 1 | 4 | 8 |
| KICH | 65 | 2 | 1 | 37 | 12 | 12 | 0 | 1 |
| KIRC | 535 | 11 | 20 | 440 | 26 | 3 | 13 | 22 |
| KIRP | 289 | 11 | 9 | 182 | 67 | 4 | 4 | 12 |
| LAML | 151 | 0 | 0 | 0 | 0 | 0 | 0 | 151 |
| LGG | 529 | 7 | 10 | 13 | 146 | 332 | 1 | 20 |
| LIHC | 374 | 32 | 52 | 133 | 142 | 1 | 2 | 12 |
| LUAD | 526 | 89 | 148 | 171 | 21 | 0 | 27 | 70 |
| LUSC | 501 | 268 | 177 | 19 | 7 | 0 | 15 | 15 |
| MESO | 86 | 30 | 20 | 9 | 13 | 0 | 10 | 4 |
| OV | 379 | 38 | 139 | 2 | 53 | 1 | 1 | 145 |
| PAAD | 178 | 50 | 36 | 43 | 1 | 1 | 19 | 28 |
| PCPG | 183 | 2 | 4 | 106 | 57 | 6 | 2 | 6 |
| PRAD | 499 | 40 | 23 | 292 | 47 | 1 | 0 | 96 |
| READ | 167 | 120 | 18 | 14 | 2 | 1 | 1 | 11 |
| SARC | 263 | 63 | 43 | 42 | 54 | 2 | 19 | 40 |
| SKCM | 471 | 41 | 26 | 15 | 15 | 3 | 3 | 368 |
| STAD | 375 | 116 | 183 | 35 | 11 | 0 | 7 | 23 |
| TGCT | 156 | 43 | 96 | 5 | 4 | 0 | 1 | 7 |
| THCA | 510 | 7 | 18 | 445 | 23 | 2 | 2 | 13 |
| THYM | 119 | 0 | 0 | 0 | 0 | 0 | 0 | 119 |
| UCEC | 548 | 247 | 201 | 52 | 23 | 1 | 1 | 23 |
| UCS | 56 | 38 | 15 | 0 | 2 | 0 | 1 | 0 |
| UVM | 80 | 2 | 1 | 29 | 46 | 2 | 0 | 0 |
| Total | 10327 | 2376 | 2517 | 2385 | 1144 | 380 | 180 | 1345 |
