# Supplementary material for: Pan-cancer analysis of the FAM83 family and its association with prognosis and tumor microenvironment
Source: Front Genet. 2022 Jul 22;13:919559. doi: 10.3389/fgene.2022.919559 (PMC9353330; doi:10.3389/fgene.2022.919559)
Supplement: Supplementary file 2 [file Presentation1.PPT]

## Slide 1
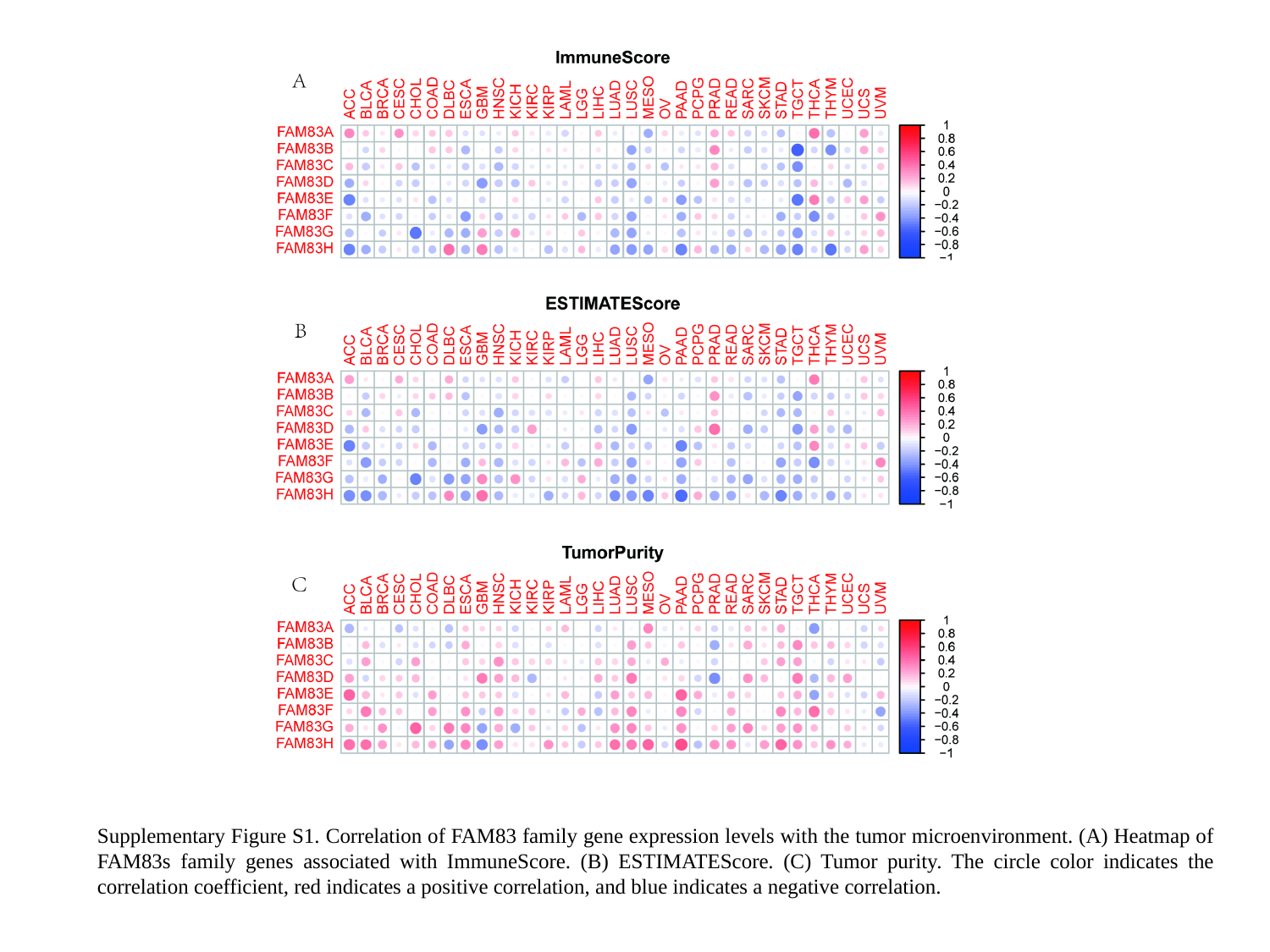

Supplementary Figure S1. Correlation of FAM83 family gene expression levels with the tumor microenvironment. (A) Heatmap of FAM83s family genes associated with ImmuneScore. (B) ESTIMATEScore. (C) Tumor purity. The circle color indicates the correlation coefficient, red indicates a positive correlation, and blue indicates a negative correlation.
